# *rac*-1-[6-Hy­droxy-4-(4-meth­oxy­phen­yl)-3,6-dimethyl-4,5,6,7-tetra­hydro-2*H*-indazol-5-yl]ethanone

**DOI:** 10.1107/S1600536813000937

**Published:** 2013-01-16

**Authors:** Konstantin A. Potekhin, Rizvan K. Askerov, Kushvar E. Hajiyeva, Narmina A. Gadirova, Shahkaram I. Nazarov

**Affiliations:** a Vladimir State University, Qor’ky St 87, 600000 Vladimir, Russian Federation; b Baku State University, Z. Khalilov St 23, AZ-1148 Baku, Azerbaijan

## Abstract

The title compound, C_18_H_22_N_2_O_3_, represents a (4*S*,5*R*,6*S*)-stereoisomer, crystallizing as a racemate in a centrosymmetric space group. The six-membered aliphatic ring adopts a half-chair conformation, with the hy­droxy- and acetyl-substituted C atoms deviating by 0.458 (2) and −0.366 (2) Å, respectively, from the plane defined by other four ring atoms. The pyrazole ring is essentially planar [r.m.s deviation = 0.004 (2) Å]. In the crystal, the mol­ecules are linked into chains along the *b* axis by N—H⋯N hydrogen bonds. The chains are linked by O—H⋯N hydrogen bonds into layers parallel to the *bc* plane.

## Related literature

For background to the use of β-cyclo­ketols as synthons in the synthesis of pyrazoles, see: Pramula *et al.* (1985 ▶). For hydrogen-bond motifs, see: Bernstein *et al.* (1995 ▶). For ring conformations, see: Cremer & Pople (1975 ▶).
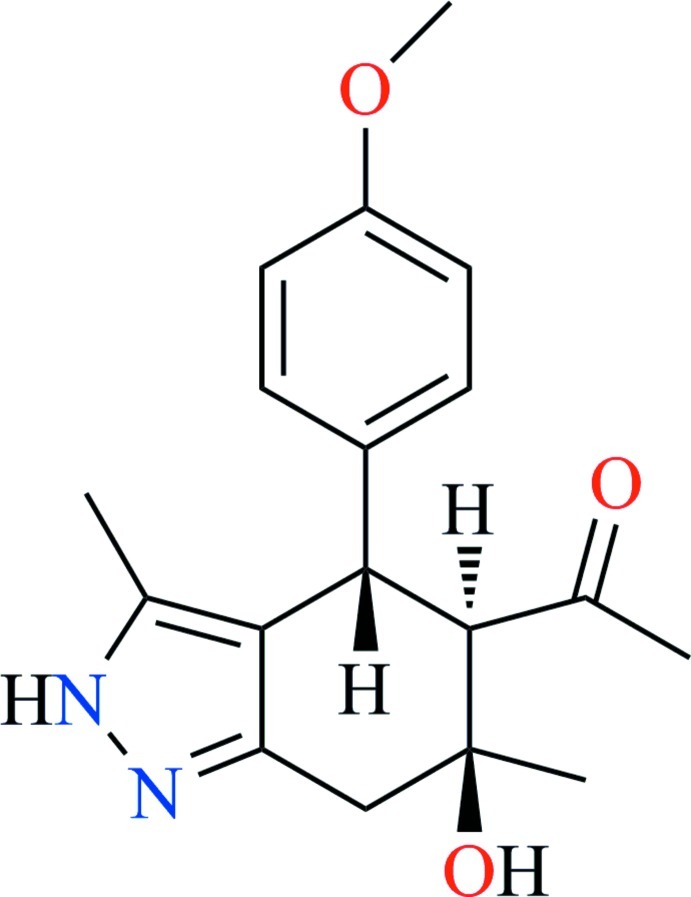


## Experimental

### 

#### Crystal data

C_18_H_22_N_2_O_3_
*M* *_r_* = 314.38
Monoclinic, 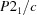

*a* = 18.3693 (14) Å
*b* = 5.6971 (4) Å
*c* = 16.3049 (12) Å
β = 109.526 (1)°
*V* = 1608.2 (2) Å^3^
*Z* = 4
Mo *K*α radiation
μ = 0.09 mm^−1^
*T* = 296 K
0.30 × 0.20 × 0.20 mm

#### Data collection

Bruker APEXII CCD diffractometer
Absorption correction: multi-scan (*SADABS*; Sheldrick, 2003 ▶) *T*_min_ = 0.974, *T*_max_ = 0.982
17994 measured reflections
4015 independent reflections
2740 reflections with *I* > 2σ(*I*)
*R*_int_ = 0.053

#### Refinement

*R*[*F* ^2^ > 2σ(*F* ^2^)] = 0.057
*wR*(*F* ^2^) = 0.151
*S* = 1.00
4015 reflections
220 parameters
H atoms treated by a mixture of independent and constrained refinement
Δρ_max_ = 0.28 e Å^−3^
Δρ_min_ = −0.26 e Å^−3^

### 

Data collection: *APEX2* (Bruker, 2005 ▶); cell refinement: *SAINT-Plus* (Bruker, 2001 ▶); data reduction: *SAINT-Plus*; program(s) used to solve structure: *SHELXTL* (Sheldrick, 2008 ▶); program(s) used to refine structure: *SHELXTL*; molecular graphics: *SHELXTL*; software used to prepare material for publication: *SHELXTL*.

## Supplementary Material

Crystal structure: contains datablock(s) global, I. DOI: 10.1107/S1600536813000937/ld2089sup1.cif

Structure factors: contains datablock(s) I. DOI: 10.1107/S1600536813000937/ld2089Isup2.hkl

Supplementary material file. DOI: 10.1107/S1600536813000937/ld2089Isup3.cml

Additional supplementary materials:  crystallographic information; 3D view; checkCIF report

## Figures and Tables

**Table 1:** Hydrogen-bond geometry (Å, °)

*D*—H⋯*A*	*D*—H	H⋯*A*	*D*⋯*A*	*D*—H⋯*A*
O1—H1*O*⋯N2^i^	0.81 (3)	2.15 (3)	2.948 (2)	168 (2)
N1—H1*N*⋯N2^ii^	0.87 (2)	2.27 (2)	3.093 (2)	157 (2)

Symmetry codes: (i) 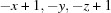; (ii) 
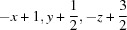
.

**Fig. 1.**
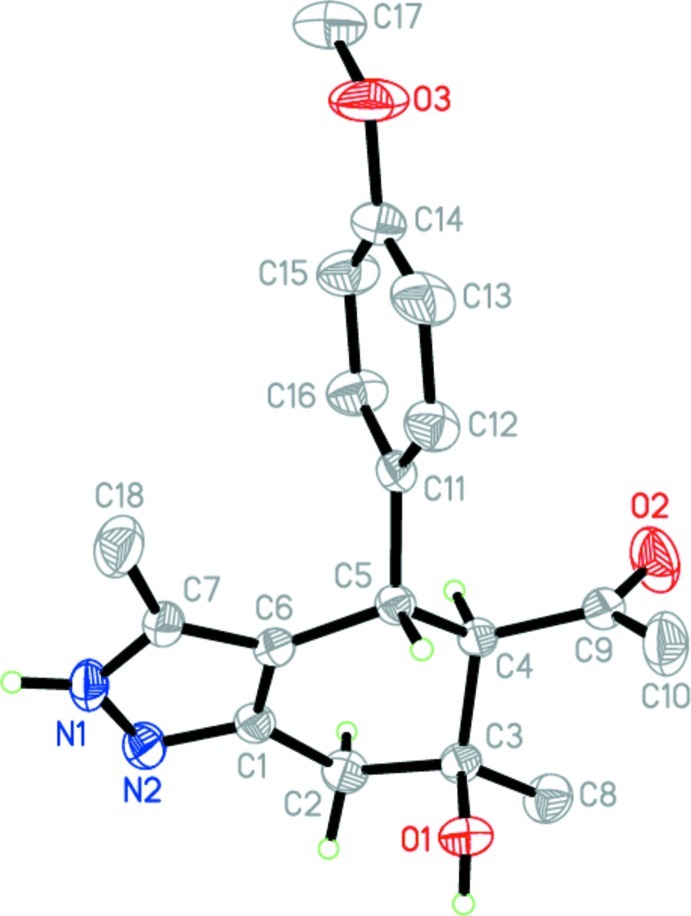
The molecular structure of the title compound with the atomic numbering scheme. Non-essential H-atoms were removed for clarity. Displacement ellipsoids were drawn at 50% probability.

**Fig. 2.**
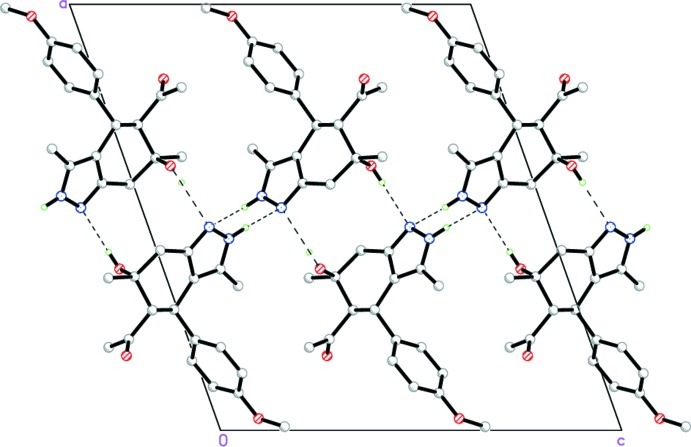
The projection of the crystal structure (I) along the b axis. H bonds are shown as a dashed lines. H atoms not involved in the formation of the hydrogen bonds are not shown.

## supplementary crystallographic information

### Comment

We explore the use of simple molecules with different functionalities for synthesis of heterocycles. Particularly, the β-cycloketols have been used as an effective synthon in syntheses of pyrazoles (Pramula *et al.* 1985).

Fig. 1 shows the molecular structure of title compound (I), C_18_H_22_N_2_O_3_. The six-membered ring C_1_, C_2_, C_3_, C_4_, C_5_, C_6_ has a half-chair conformation. Four atoms of a six-membered ring C_1_,*C*_2_, C_5_, C_6_ are located on the same plane within 0.004 (2) Å while C_3_ (+ 0.458 (2) Å) and C_4_ (- 0.366 (2) Å) atoms are deviating to the opposite sides of the plane. The pyrazole ring is essentially planar (r.m.s deviation is 0.004 (2) Å). Hydrogen bonds N—H ··· N combine molecules into chains oriented along the axis *b* (Fig.2). H-bonds O—H ··· N form centrosymmetric cycles. Through these cycles, the above chains are combined into two-dimensional layers parallel to the plane *bc*.

### Experimental

2,4-Diacetyl-5-hydroxy-5-metyl-3(4-methoxyphenyl)cyclohexanone (20 mmol) and hydrazine hydrate (20 mmol) were dissolved in 20 ml of ethanol. The mixture was stirred at 335–340 K for 10 h. After cooling to the room temperature, white crystals were obtained. The crystals were filtered off and washed with cold ethanol. Then they were dissolved in ethanol (50 ml) and recrystallized to yield colourless block-shaped crystals suitable for data collection.

### Refinement

Hydrogen atoms of the OH and NH group found in difference-Fourier maps and included in the refinement with isotropic displacement parameters. The other hydrogen atoms were placed in calculated positions with and refined in the riding mode with isotropic displacement parameters restricted to 1.2*U*_eq_ of the adjacent C atom (1.5*U*_eq_ for methyl C atoms).

### Crystal data

**Table:**

C_18_H_22_N_2_O_3_	*F*(000) = 672
*M**_r_* = 314.38	*D*_x_ = 1.298 Mg m^−^^3^
Monoclinic, *P*2_1_/*c*	Mo *K*α radiation, λ = 0.71073 Å
Hall symbol: -P 2ybc	Cell parameters from 2645 reflections
*a* = 18.3693 (14) Å	θ = 2.4–27.8°
*b* = 5.6971 (4) Å	µ = 0.09 mm^−^^1^
*c* = 16.3049 (12) Å	*T* = 296 K
β = 109.526 (1)°	Prism, yellow
*V* = 1608.2 (2) Å^3^	0.30 × 0.20 × 0.20 mm
*Z* = 4	

### Data collection

**Table:**

Bruker APEXII CCD diffractometer	4015 independent reflections
Radiation source: fine-focus sealed tube	2740 reflections with *I* > 2σ(*I*)
Graphite monochromator	*R*_int_ = 0.053
φ and ω scans	θ_max_ = 28.4°, θ_min_ = 2.4°
Absorption correction: multi-scan (*SADABS*; Sheldrick, 2003)	*h* = −24→24
*T*_min_ = 0.974, *T*_max_ = 0.982	*k* = −7→7
17994 measured reflections	*l* = −21→21

### Refinement

**Table:**

Refinement on *F*^2^	Primary atom site location: structure-invariant direct methods
Least-squares matrix: full	Secondary atom site location: difference Fourier map
*R*[*F*^2^ > 2σ(*F*^2^)] = 0.057	Hydrogen site location: difference Fourier map
*wR*(*F*^2^) = 0.151	H atoms treated by a mixture of independent and constrained refinement
*S* = 1.00	*w* = 1/[σ^2^(*F*_o_^2^) + (0.0775*P*)^2^ + 0.4526*P*] where *P* = (*F*_o_^2^ + 2*F*_c_^2^)/3
4015 reflections	(Δ/σ)_max_ < 0.001
220 parameters	Δρ_max_ = 0.28 e Å^−^^3^
0 restraints	Δρ_min_ = −0.26 e Å^−^^3^

### Special details

**Table:**

Geometry. All e.s.d.'s (except the e.s.d. in the dihedral angle between two l.s. planes) are estimated using the full covariance matrix. The cell e.s.d.'s are taken into account individually in the estimation of e.s.d.'s in distances, angles and torsion angles; correlations between e.s.d.'s in cell parameters are only used when they are defined by crystal symmetry. An approximate (isotropic) treatment of cell e.s.d.'s is used for estimating e.s.d.'s involving l.s. planes.
Refinement. Refinement of *F*^2^ against ALL reflections. The weighted *R*-factor *wR* and goodness of fit *S* are based on *F*^2^, conventional *R*-factors *R* are based on *F*, with *F* set to zero for negative *F*^2^. The threshold expression of *F*^2^ > σ(*F*^2^) is used only for calculating *R*-factors(gt) *etc*. and is not relevant to the choice of reflections for refinement. *R*-factors based on *F*^2^ are statistically about twice as large as those based on *F*, and *R*- factors based on ALL data will be even larger.

### Fractional atomic coordinates and isotropic or equivalent isotropic displacement parameters (Å^2^)

**Table:**

	*x*	*y*	*z*	*U*_iso_*/*U*_eq_	
O1	0.37902 (8)	0.0325 (2)	0.39112 (9)	0.0292 (3)	
H1O	0.4175 (15)	0.014 (4)	0.3782 (15)	0.042 (7)*	
O2	0.17526 (9)	−0.3321 (3)	0.33143 (10)	0.0509 (5)	
O3	0.02786 (9)	0.2338 (3)	0.60541 (12)	0.0581 (5)	
N1	0.44992 (10)	0.1360 (3)	0.68991 (11)	0.0323 (4)	
H1N	0.4757 (13)	0.189 (4)	0.7417 (15)	0.036 (6)*	
N2	0.47720 (9)	−0.0401 (3)	0.65158 (10)	0.0317 (4)	
C1	0.41943 (10)	−0.0765 (3)	0.57672 (11)	0.0250 (4)	
C2	0.42242 (11)	−0.2495 (3)	0.50915 (12)	0.0291 (4)	
H2B	0.4132	−0.4062	0.5267	0.035*	
H2C	0.4732	−0.2469	0.5033	0.035*	
C3	0.36087 (10)	−0.1881 (3)	0.42137 (11)	0.0240 (4)	
C4	0.28224 (10)	−0.1512 (3)	0.43618 (11)	0.0233 (4)	
H4A	0.2756	−0.2845	0.4710	0.028*	
C5	0.28328 (10)	0.0725 (3)	0.49055 (11)	0.0224 (4)	
H5A	0.2854	0.2083	0.4545	0.027*	
C6	0.35664 (10)	0.0713 (3)	0.56806 (11)	0.0236 (4)	
C7	0.37853 (10)	0.2084 (3)	0.64195 (12)	0.0274 (4)	
C8	0.35771 (13)	−0.3796 (4)	0.35532 (13)	0.0359 (5)	
H8A	0.4077	−0.3967	0.3494	0.054*	
H8B	0.3206	−0.3377	0.3001	0.054*	
H8C	0.3428	−0.5252	0.3748	0.054*	
C9	0.21385 (11)	−0.1556 (4)	0.35177 (12)	0.0296 (4)	
C10	0.19493 (14)	0.0554 (4)	0.29457 (13)	0.0429 (5)	
H10A	0.1628	0.0102	0.2372	0.064*	
H10B	0.2418	0.1242	0.2919	0.064*	
H10C	0.1681	0.1676	0.3178	0.064*	
C11	0.21160 (10)	0.0998 (3)	0.51714 (11)	0.0241 (4)	
C12	0.16362 (11)	0.2925 (4)	0.49013 (13)	0.0327 (4)	
H12A	0.1726	0.3980	0.4511	0.039*	
C13	0.10256 (12)	0.3313 (4)	0.52006 (15)	0.0404 (5)	
H13A	0.0713	0.4625	0.5014	0.048*	
C14	0.08796 (11)	0.1756 (4)	0.57763 (14)	0.0366 (5)	
C15	0.13375 (13)	−0.0199 (4)	0.60398 (15)	0.0406 (5)	
H15A	0.1237	−0.1274	0.6418	0.049*	
C16	0.19500 (12)	−0.0552 (4)	0.57368 (14)	0.0368 (5)	
H16A	0.2259	−0.1873	0.5920	0.044*	
C17	0.00859 (16)	0.0772 (6)	0.66259 (19)	0.0634 (8)	
H17A	−0.0343	0.1391	0.6768	0.095*	
H17B	0.0522	0.0593	0.7149	0.095*	
H17C	−0.0051	−0.0727	0.6348	0.095*	
C18	0.33930 (13)	0.4034 (4)	0.67129 (14)	0.0416 (5)	
H18A	0.3487	0.3891	0.7326	0.062*	
H18B	0.2847	0.3962	0.6406	0.062*	
H18C	0.3591	0.5510	0.6596	0.062*	

### Atomic displacement parameters (Å^2^)

**Table:**

	*U*^11^	*U*^22^	*U*^33^	*U*^12^	*U*^13^	*U*^23^
O1	0.0287 (7)	0.0281 (7)	0.0358 (7)	0.0006 (6)	0.0173 (6)	0.0041 (6)
O2	0.0481 (10)	0.0498 (10)	0.0436 (9)	−0.0213 (8)	0.0006 (7)	−0.0046 (8)
O3	0.0388 (9)	0.0800 (13)	0.0677 (11)	0.0212 (9)	0.0342 (8)	0.0121 (10)
N1	0.0282 (9)	0.0422 (10)	0.0218 (8)	0.0005 (7)	0.0023 (7)	−0.0050 (7)
N2	0.0244 (8)	0.0415 (10)	0.0266 (8)	0.0043 (7)	0.0049 (7)	0.0006 (7)
C1	0.0211 (9)	0.0299 (10)	0.0240 (9)	0.0020 (7)	0.0074 (7)	0.0029 (7)
C2	0.0266 (9)	0.0295 (10)	0.0316 (10)	0.0059 (8)	0.0101 (8)	0.0004 (8)
C3	0.0249 (9)	0.0237 (9)	0.0245 (9)	0.0008 (7)	0.0096 (7)	−0.0002 (7)
C4	0.0229 (9)	0.0255 (9)	0.0217 (8)	−0.0025 (7)	0.0074 (7)	0.0017 (7)
C5	0.0199 (8)	0.0247 (9)	0.0220 (8)	0.0016 (7)	0.0063 (7)	0.0022 (7)
C6	0.0225 (9)	0.0277 (9)	0.0218 (8)	0.0003 (7)	0.0087 (7)	0.0003 (7)
C7	0.0255 (9)	0.0339 (10)	0.0218 (9)	0.0008 (8)	0.0067 (7)	0.0001 (8)
C8	0.0416 (12)	0.0330 (11)	0.0361 (11)	−0.0005 (9)	0.0169 (9)	−0.0083 (9)
C9	0.0264 (9)	0.0381 (11)	0.0257 (9)	−0.0038 (8)	0.0106 (8)	−0.0045 (8)
C10	0.0413 (12)	0.0506 (14)	0.0284 (10)	−0.0003 (10)	0.0006 (9)	0.0047 (10)
C11	0.0196 (8)	0.0281 (9)	0.0227 (8)	0.0012 (7)	0.0045 (7)	−0.0006 (7)
C12	0.0287 (10)	0.0326 (11)	0.0360 (10)	0.0038 (8)	0.0098 (8)	0.0062 (9)
C13	0.0284 (10)	0.0418 (12)	0.0500 (13)	0.0158 (9)	0.0120 (9)	0.0071 (10)
C14	0.0247 (10)	0.0484 (13)	0.0388 (11)	0.0053 (9)	0.0133 (9)	−0.0028 (10)
C15	0.0384 (12)	0.0461 (13)	0.0440 (12)	0.0059 (9)	0.0227 (10)	0.0142 (10)
C16	0.0358 (11)	0.0355 (11)	0.0439 (12)	0.0133 (9)	0.0198 (9)	0.0129 (9)
C17	0.0423 (14)	0.094 (2)	0.0652 (17)	−0.0028 (14)	0.0330 (13)	−0.0015 (16)
C18	0.0427 (12)	0.0475 (13)	0.0321 (11)	0.0069 (10)	0.0091 (9)	−0.0116 (10)

### Geometric parameters (Å, º)

**Table:**

O1—C3	1.430 (2)	C8—H8A	0.9600
O1—H1O	0.81 (3)	C8—H8B	0.9600
O2—C9	1.211 (2)	C8—H8C	0.9600
O3—C14	1.367 (2)	C9—C10	1.489 (3)
O3—C17	1.418 (3)	C10—H10A	0.9600
N1—C7	1.348 (2)	C10—H10B	0.9600
N1—N2	1.363 (2)	C10—H10C	0.9600
N1—H1N	0.87 (2)	C11—C16	1.382 (3)
N2—C1	1.339 (2)	C11—C12	1.385 (3)
C1—C6	1.397 (2)	C12—C13	1.382 (3)
C1—C2	1.493 (3)	C12—H12A	0.9300
C2—C3	1.539 (3)	C13—C14	1.382 (3)
C2—H2B	0.9700	C13—H13A	0.9300
C2—H2C	0.9700	C14—C15	1.375 (3)
C3—C8	1.520 (3)	C15—C16	1.386 (3)
C3—C4	1.556 (2)	C15—H15A	0.9300
C4—C9	1.522 (2)	C16—H16A	0.9300
C4—C5	1.549 (2)	C17—H17A	0.9600
C4—H4A	0.9800	C17—H17B	0.9600
C5—C6	1.508 (2)	C17—H17C	0.9600
C5—C11	1.525 (2)	C18—H18A	0.9600
C5—H5A	0.9800	C18—H18B	0.9600
C6—C7	1.378 (3)	C18—H18C	0.9600
C7—C18	1.488 (3)		
			
C3—O1—H1O	107.3 (17)	C3—C8—H8C	109.5
C14—O3—C17	118.17 (19)	H8A—C8—H8C	109.5
C7—N1—N2	113.26 (16)	H8B—C8—H8C	109.5
C7—N1—H1N	124.4 (15)	O2—C9—C10	120.39 (18)
N2—N1—H1N	122.3 (15)	O2—C9—C4	119.46 (18)
C1—N2—N1	103.36 (15)	C10—C9—C4	120.14 (17)
N2—C1—C6	112.02 (16)	C9—C10—H10A	109.5
N2—C1—C2	124.16 (16)	C9—C10—H10B	109.5
C6—C1—C2	123.80 (16)	H10A—C10—H10B	109.5
C1—C2—C3	109.87 (14)	C9—C10—H10C	109.5
C1—C2—H2B	109.7	H10A—C10—H10C	109.5
C3—C2—H2B	109.7	H10B—C10—H10C	109.5
C1—C2—H2C	109.7	C16—C11—C12	117.36 (17)
C3—C2—H2C	109.7	C16—C11—C5	121.89 (16)
H2B—C2—H2C	108.2	C12—C11—C5	120.58 (17)
O1—C3—C8	110.14 (14)	C13—C12—C11	121.33 (19)
O1—C3—C2	109.69 (15)	C13—C12—H12A	119.3
C8—C3—C2	109.78 (15)	C11—C12—H12A	119.3
O1—C3—C4	105.66 (14)	C12—C13—C14	120.12 (19)
C8—C3—C4	112.92 (15)	C12—C13—H13A	119.9
C2—C3—C4	108.54 (14)	C14—C13—H13A	119.9
C9—C4—C5	112.31 (15)	O3—C14—C15	124.8 (2)
C9—C4—C3	112.70 (14)	O3—C14—C13	115.54 (19)
C5—C4—C3	111.46 (14)	C15—C14—C13	119.67 (18)
C9—C4—H4A	106.6	C14—C15—C16	119.4 (2)
C5—C4—H4A	106.6	C14—C15—H15A	120.3
C3—C4—H4A	106.6	C16—C15—H15A	120.3
C6—C5—C11	112.13 (14)	C11—C16—C15	122.11 (18)
C6—C5—C4	108.23 (14)	C11—C16—H16A	118.9
C11—C5—C4	113.58 (14)	C15—C16—H16A	118.9
C6—C5—H5A	107.5	O3—C17—H17A	109.5
C11—C5—H5A	107.5	O3—C17—H17B	109.5
C4—C5—H5A	107.5	H17A—C17—H17B	109.5
C7—C6—C1	105.28 (16)	O3—C17—H17C	109.5
C7—C6—C5	130.39 (16)	H17A—C17—H17C	109.5
C1—C6—C5	124.31 (16)	H17B—C17—H17C	109.5
N1—C7—C6	106.07 (16)	C7—C18—H18A	109.5
N1—C7—C18	121.61 (17)	C7—C18—H18B	109.5
C6—C7—C18	132.32 (17)	H18A—C18—H18B	109.5
C3—C8—H8A	109.5	C7—C18—H18C	109.5
C3—C8—H8B	109.5	H18A—C18—H18C	109.5
H8A—C8—H8B	109.5	H18B—C18—H18C	109.5
			
C7—N1—N2—C1	−0.1 (2)	N2—N1—C7—C6	0.7 (2)
N1—N2—C1—C6	−0.6 (2)	N2—N1—C7—C18	−178.36 (18)
N1—N2—C1—C2	177.76 (17)	C1—C6—C7—N1	−1.0 (2)
N2—C1—C2—C3	−160.60 (17)	C5—C6—C7—N1	−179.50 (18)
C6—C1—C2—C3	17.5 (2)	C1—C6—C7—C18	178.0 (2)
C1—C2—C3—O1	65.31 (18)	C5—C6—C7—C18	−0.6 (4)
C1—C2—C3—C8	−173.54 (15)	C5—C4—C9—O2	−132.80 (19)
C1—C2—C3—C4	−49.68 (19)	C3—C4—C9—O2	100.3 (2)
O1—C3—C4—C9	78.02 (18)	C5—C4—C9—C10	47.5 (2)
C8—C3—C4—C9	−42.4 (2)	C3—C4—C9—C10	−79.4 (2)
C2—C3—C4—C9	−164.39 (15)	C6—C5—C11—C16	−57.2 (2)
O1—C3—C4—C5	−49.32 (17)	C4—C5—C11—C16	65.9 (2)
C8—C3—C4—C5	−169.78 (15)	C6—C5—C11—C12	117.95 (19)
C2—C3—C4—C5	68.27 (18)	C4—C5—C11—C12	−118.97 (19)
C9—C4—C5—C6	−174.75 (14)	C16—C11—C12—C13	1.4 (3)
C3—C4—C5—C6	−47.19 (18)	C5—C11—C12—C13	−173.94 (19)
C9—C4—C5—C11	60.06 (19)	C11—C12—C13—C14	−0.4 (3)
C3—C4—C5—C11	−172.39 (14)	C17—O3—C14—C15	−3.0 (4)
N2—C1—C6—C7	1.0 (2)	C17—O3—C14—C13	178.2 (2)
C2—C1—C6—C7	−177.33 (17)	C12—C13—C14—O3	178.0 (2)
N2—C1—C6—C5	179.63 (16)	C12—C13—C14—C15	−1.0 (3)
C2—C1—C6—C5	1.3 (3)	O3—C14—C15—C16	−177.5 (2)
C11—C5—C6—C7	−42.2 (3)	C13—C14—C15—C16	1.3 (4)
C4—C5—C6—C7	−168.21 (18)	C12—C11—C16—C15	−1.0 (3)
C11—C5—C6—C1	139.57 (18)	C5—C11—C16—C15	174.3 (2)
C4—C5—C6—C1	13.5 (2)	C14—C15—C16—C11	−0.3 (4)

### Hydrogen-bond geometry (Å, º)

**Table:**

*D*—H···*A*	*D*—H	H···*A*	*D*···*A*	*D*—H···*A*
O1—H1*O*···N2^i^	0.81 (3)	2.15 (3)	2.948 (2)	168 (2)
N1—H1*N*···N2^ii^	0.87 (2)	2.27 (2)	3.093 (2)	157 (2)

Symmetry codes: (i) −*x*+1, −*y*, −*z*+1; (ii) −*x*+1, *y*+1/2, −*z*+3/2.
